# Reuse of coir, peat, and wood fiber in strawberry production

**DOI:** 10.3389/fpls.2023.1307240

**Published:** 2024-01-12

**Authors:** Tomasz Woznicki, Krzysztof Kusnierek, Bart Vandecasteele, Anita Sønsteby

**Affiliations:** ^1^ Department of Horticulture, Norwegian Institute of Bioeconomy Research (NIBIO), Kapp, Norway; ^2^ Department of Agricultural Technology and System Analysis, Center for Precision Agriculture, Norwegian Institute of Bioeconomy Research (NIBIO), Kapp, Norway; ^3^ Plant Sciences Unit, Flanders Research Institute for Agriculture, Fisheries and Food (ILVO), Melle, Belgium

**Keywords:** strawberries (*Fragaria* x *ananassa*), growing media, sustainability, circular economy, soilless culture system, substrate properties, nutrient recycling

## Abstract

**Introduction:**

Production of strawberries in greenhouses and polytunnels is gaining popularity worldwide. This study investigated the effect of reuse of coir and peat, two substrates commonly adapted to soilless strawberry production, as well as stand-alone wood fiber from Norway spruce, a promising substrate candidate.

**Methods:**

The experiment was performed in a polytunnel at NIBIO Apelsvoll, Norway, and evaluated both virgin substrates, as well as spent materials that were used in one or two years. Yield, berry quality and plant architecture of the strawberry cultivar ‘Malling Centenary’ were registered. In addition, chemical and physical properties of virgin and reused substrates were investigated.

**Results:**

While plants grown in peat and wood fiber had highest yield in the first year of production, the berry yield was slightly reduced when these substrates were utilized for the second and third time. However, yield was comparable to the yield level attained in new and reused coir. Interestingly, berries grown in wood fiber had a tendency to a higher sugar accumulation. This substrate also produced the highest plants. Stand-alone wood fiber was the substrate with the highest accumulation of nitrogen during the three consecutive production cycles. All three investigated materials revealed a trend for decreased potassium accumulation. Wood fiber is characterized by the highest percentage of cellulose, however after three years of production the cellulose content was reducedto the same levels as for coir and peat.

**Discussion:**

Implementation of wood fiber as a growing medium, as well as general practice of substrate reuse can be therefore an achievable strategy for more sustainable berry production.

## 1 Introduction

The world population is projected to reach 8.5 billion in 2030, and to increase further to 9.7 billion in 2050 and to 10.4 billion by 2100 (UN, Department of Economic and Social Affairs, Population Division, 2019). To be able to feed the global population in the future and maintain sustainability of the food production systems, substantial improvements in agricultural and horticultural practices are needed. A broader implementation of soilless culture systems (SCS) will enhance productivity and reduce negative environmental impact of plant production by improving water and nutrient use efficiency (Gruda, 2019). In this type of production, ‘growing media’ or ‘horticultural substrates’ provide a root environment that ensures optimal water and nutrient supply and adequate aeration. Since SCS popularity is increasing worldwide, the global growing media demand is predicted to be four times higher in the next 30 years (Blok et al., 2021).

Strawberry is one of the most important berry crops globally, and its production is increasing (FAOSTAT, 2020; Hernández-Martínez et al., 2023). Peat and coir are commonly used substrates in tunnel and greenhouse strawberry production; however, use of these materials has a negative environmental impact (Gruda, 2019). Peatlands are important carbon storage and water retention reservoirs which are only partially renewable. Therefore, policy regulations and customer preferences are becoming more restrictive toward the use of peat and peat-based substrates. Similarly, one of the most popular alternatives for peat, coconut coir, has several drawbacks, for example: environmental degradation due to land use change during coir production and water pollution during the coir processing. In addition, the material is produced in Asia, and the high CO_2_ footprint due to the long transportation is an issue from a European or American perspective. Many regions where coconuts are grown, including previously forested areas, have experienced deforestation due to coconut cultivation. This is particularly evident in places like the Pacific Islands, where coconuts are a major contributor to deforestation (Kumar and Kunhamu, 2022). SCS possess a plasticity potential to utilize organic by-products from other industries, for example forestry (Barrett et al., 2016). Therefore, conifer wood fiber became recently a promising, more sustainable alternative, mainly due to the fact that the natural forest habitats are not changed because of straightforward reforestation. According to the Quantis (2012), production, use and disposal of wood fiber releases only 75 kg CO_2_ equivalents per 1 m3, whereas for peat, 300 kg CO_2_ equivalents per 1 m3 are released. The material has been tested as a growing media ingredient and was recently applied also as a stand-alone substrate for strawberry cultivation (Woznicki et al., 2021; Aurdal et al., 2023; Woznicki et al., 2023).

The disposal of spent substrates at the end of cultivation is a known threat to the environment (Incrocci et al., 2012). Even though some types of spent growing media can be directly recycled as a soil improver, either incorporated into composting process as a bulking agent (Viaene et al., 2017), or as other materials, like Miscanthus, can be used as a solid fuel or biochar precursors (Kraska et al., 2018), there is a substantial risk that growing media may contain harmful chemicals and/or nutrients which can directly affect the ecosystems. In addition, growing media disposed to landfill at the end of the growing cycle generate additional costs and recently, landfill availability is becoming more and more reduced (Ahire et al., 2022).

Since sustainability has recently become a major concern, minimizing the environmental impact of horticultural production is an important topic among researchers and policy makers. Therefore, it is suggested that the circular economy concept of ‘3R’ (Reduce, Reuse and Recycle) should be applied to the entire life cycle of growing media used for both hobby and professional horticulture (Fiorello et al., 2023). In accordance with the Directive EU2018/851 of the European Parliament and of The Council, “waste management in the European Union should be improved and transformed into sustainable material management, with a view to protecting, preserving, and improving the quality of the environment, protecting human health, ensuring prudent, efficient, and rational utilization of natural resources, promoting the principles of the circular economy” (EU, 2018).

In recent years, the reuse of growing substrates has gained increasing attention due to its potential economic and environmental benefits. Recchia et al. (2013) concluded that reuse of the spent growing medium has less environmental impact when compared to landfilling. However, reuse of substrates may reduce their quality. Accumulation of nutrients may occur in organic substrates during a growing season and their subsequent reuse may lead to plant stress and production losses. The applicability of reused substrates depends on the physio-chemical properties of the material as well as on the crop specific tolerance for unfavorable conditions in the root zone (Incrocci et al., 2012).

Strawberries grown in SCS have relatively high nutritional requirements. Under optimal growth conditions, the strawberry plant has the highest demand for potassium, followed by nitrogen, calcium, magnesium, and phosphorus, making the fruits especially rich in N, P and K (Tagliavini et al., 2005). Moreover, it was observed that in multi-seasonal strawberry production, approximately 40% of the N stored in the plants was remobilized during the plant regrowth in the spring (Tagliavini et al., 2005). Therefore, it is important to provide adequate mineral nutrition not only during the production phase but also in the period of plant establishment. The nutritional composition of growing medium in this period may play an important role for further biomass production and realizing of the yield potential. Use of a growing media in a given season may lead to changes of the physical and chemical properties and the reuse in subsequent growing seasons may stimulate or suppress plant establishment and crop production. However, such effects are not fully understood.

To address all these issues, the objective of this study was to evaluate the effects of reuse of coir, peat and wood fiber in three production cycles, identify changes in their physical and chemical composition and record growth, yield and quality parameters of tunnel-grown strawberries.

## 2 Materials and methods

### 2.1 Plant cultivation

Tray plants of the June bearing strawberry cultivar ‘Malling Centenary’ (*Fragaria* x *ananassa*) were grown in three different substrates in a high poly-tunnel (Haygrove Gothic, oriented South-North) on a table-top system at the NIBIO research station Apelsvoll, Kapp, Norway (60°40′ N), during three consecutive years and under the same agrochemical conditions.

The following growing media were used in the experiment:

-100% coir (Botanicoir Precision Plus Ultra, UK), (**C**)

-80% peat (H2-H4, limed, Tjerbo, Norway) and 20% perlite [Agra-perlite, Pull Rhenen, NL (Grade 3 – 0-6.5mm)], (V/V), (**P**)

-100% Norway spruce wood fiber, coarse (Fibergrow, Hunton Fiber AS, Norway), (**WF**)

Each year, the plants were acquired from NORGRO AS, Norway, as cold-stored tray (plug) plants and planted in 8 L plastic trays (length = 50 cm, width = 18 cm, height of substrate = 14 cm) with four plants in each tray and with two trays representing one replicate (8 plants per replicate). After the first and second year of production the above-ground plant biomass was removed, and the substrates were stored under the roof of unheated plastic tunnel, to be reused in the following year. The substrates dried during winter and were rewetted before use the next growing season. To simulate real farming conditions and minimize cost of operation, the old plug/root from the tray plants was kept in the trays, and the new plants were planted in-between the old root plugs. The only exception was after the second year of production, when the remaining root plug of one of the old plants was removed and replaced by a new plant. Fertigation (EC 1.6 mS/cm, pH 6.0, Calcinit and Kristalon Scarlet, Yara, Norway,50%/50%, containing macronutrients:10.6; 1.0; 4.7; 0.7; 0.8; 3.2 mmol/l of N, P, K, Mg, S, Ca and micronutrients: 34; 21; 0.75; 5; 10; 0.5 μmol/L of Fe, Mn, Cu, Zn, B, Mo, respectively), was applied continuously during the entire study by one drip (1.2 L/h) per plant and timing was adjusted to the environmental conditions in the tunnel using a Priva-system sensors (Priva, ON, Canada). A detailed fertigation schedule is presented in **Table 1**. Fixed watering duration times (4 min per watering event) were applied throughout the experiment. A standard commercial plant protection strategy, including application of predatory mites and pulsed water mists against powdery mildew (Asalf et al., 2021), was successfully used to prevent pests and diseases. Runners were removed throughout the season. In the third and final year of production, the experiment included a 2x3 split-plot design involving two factors (substrate type and year of cultivation), each with three levels: three substrates (coir – C, peat – P, and wood fiber - WF), which were 1) not used before, 2) were used for one or 3) two seasons before. The results obtained in the final season are presented here, and the numbers 1, 2 and 3 included in the figures, indicate the age of the substrate at the end of the experiment as explained above.

**Table 1:** Implemented watering strategy.

Time of the day	Watering criterion
9.00-10.00	When temp. > 20°C and solar radiation > 500W/m^2^
10.00-13.00	Fixed watering at 10.00 and 12.00
13.00-17.00	When daily radiation sum > 500J/m^2^ (min. 1.5 h between watering)
17.00-21.00	When temp. > 23°C, (min. 1.5 h between watering)

Berries were harvested three times a week throughout the season and the weight of all berries collected per replicate was recorded. Here, only the yield of marketable berries is included because the proportion of the other yield fractions (rotten and exceptionally small berries) was negligible. Marketable berries representing each week of production were frozen (-20°C) and further analyzed for their chemical composition. The obtained results were further averaged and are presented as mean values representing the whole growing season. At termination of the experiment, biomass production (g FW) and plant height (cm) as well as number of crowns and leaves were recorded for all the plants in each treatment. The results are presented on a per plant basis.

### 2.2 Chemical composition of strawberries

For soluble solids and titratable acids analysis, 200 g of representative and uniform frozen fruits from each treatment and each harvest week was thawed overnight at 20°C and homogenized using a blender (Braun MR400, Karlsruhe, Germany). The samples were then filtered (Whatman 125 mm, Schleicher & Schuell, Dassel, Germany) and centrifuged at 400 rpm for 15 min (Eppendorf 5810 R, Hamburg, Germany) to obtain juice. Soluble solid concentration was determined from the juice by a digital refractometer (Atago refractometer model PR-1 CO, LTD, Tokyo, Japan), measured as Brix0 and expressed as % soluble solids. Titratable acids were determined by a radiometer endpoint titrator (Metrohm 716 DMS Titrino and 730 Sample Changer, Herisau, Switzerland) that calculated citric acid expressed as a percentage. For determination of dry matter %, berry homogenate (10 g) was dried at 100°C for 24 h in a drying oven (Termaks, Bergen, Norway) and stabilized in a desiccator before weighing.

### 2.3 Physical and chemical properties of the substrates

Properties of the substrates were analyzed after termination of the experiment. Both unused virgin materials and reused substrates (after removal of all plugs remains) were tested for its physical and chemical characteristics at ILVO, Belgium.

Methods for substrate analysis are based on European Standards developed by the European Committee for standardization (CEN) and are assigned to the European Standard EN numbers. Sample preparation of growing media for analyses were conducted according to EN13040 (CEN, 2007). To assess the dry bulk density, water on fresh and dry weight (at −10 cm, −50 cm and −100 cm, as an indicator of water holding capacity), total pore volume (at −10 cm), air and water volume % (at −10 cm, −50 cm and −100 cm), easy obtainable water, water buffering capacity, shrinkage, moisture content, dry matter content, organic matter content and ash content, the EN13039 and EN13041 (CEN, 2012) procedures were employed.

Prior to chemical analysis, each growing media sample (tray) was dried at 70°C, ground, and considered as individual biological replicates. Total nitrogen (N) content was determined using the Dumas method, EN13654–2 (CEN, 2001), and organic carbon (OC) was measured using a Skalar Primacs SNC 100 analyzer (Skalar, The Netherlands). For the assessment of total contents of phosphorus (P), potassium (K), magnesium (Mg), calcium (Ca) and sodium (Na), 0.5 g of dried and ground material was digested using a DigiPREP MS 200 Block Digestion System (SCP SCIENCE, Québec, Canada) with 4 mL HNO3 (p.a. 65%) and 12 mL HCl (p.a. 37%) for 120 minutes at 105°C. An Agilent 5110 VDV ICP-OES (Agilent, Santa Clara, CA, USA) was used to analyze the extract. Neutral detergent fibre (NDF), acid detergent fibre (ADF) and acid detergent lignin (ADL, lignin percentage) content was determined using an Ankom220 Fiber Analyzer extraction unit according to Van Soest et al. (1991). Based on NDF, ADF and ADL percentage, the following calculations were performed to obtain percentage of hemicellulose and cellulose: %hemicellulose = %NDF − %ADF, and %cellulose = %ADF − %ADL. The lignin, cellulose, hemicellulose, and residual fraction are expressed on OM base (%/OM) by conversion of the fraction expressed on DM base (%/DM) to 100% OM:100 x (fraction (%/DM))/(%OM/DM). Residual fraction (%/DM) = %OM/DM - lignin (%/DM) - cellulose (%/DM) - hemicellulose (%/DM).

The cation exchange capacity (CEC) was determined by ammonium acetate (p.a. > 99%, Chem-Lab NV) at pH 7.0 and KCl (p.a. > 99.5%, Chem-Lab NV), modified from the method by Rajkovich et al. (2012). Electrical conductivity (EC) (EN 13038) and pH-H_2_O (EN 13037) were measured in a 1:5 solid to water (v/v) suspension. As an indicator of substrate stability, oxygen uptake rate (OUR) was calculated from the oxygen consumption due to microbial activity. 20 g of substrate was mixed in 200 mL buffered nutrient solution during 5 days of shaking in a closed Oxitop respirometer based on the method reported by Grigatti et al. (2011). The materials were tested for immobilization of mineral N (Vandecasteele et al., 2018a) by adding 350 mg N/L material followed by incubation at 37°C for 7 days. CO_2_ emission was measured 13 times during 30 days using a LI-8100 Automated CO_2_ Flux System equipped with a soil flux chamber and a non-dispersive infrared gas analyzer (LI-COR Biosciences, Lincoln, NE, USA). Two liters of material was mixed with 4 g/L Haifa Multi-Mix Potting Soil 14 + 16 + 18(+micronutrients) fertilizer (MF), moistened based on the squeeze test, and put in PVC rings (height: 12 cm, diameter: 25 cm) at 20°C. The rings were closed at the bottom with a plastic cover. The mixtures were rewetted twice a week based on the recorded weight loss. The cumulative CO_2_ release after 30 days was expressed as mol CO_2_/kg OM (Vandecasteele et al., 2020). Approximation of the amount of residual macronutrients in spent growing media after each production cycle (in kg/ha) was based on assumption that one hectare of table-top strawberry production utilizes ca. 13000 half meter trays, and that each of them contains 8L of growing medium. Based on the dry bulk density of the substrates (73, 90 and 37 kg/m3 for C, P and WF, respectively) their dry matter was calculated per tray basis (0.6 kg for C, 0.7 kg for P and 0.3 kg for WF).

### 2.4 Statistical analysis

Statistical analysis for substrate properties and the plant growth trial followed the data presentation paradigm suggested by Weissgerber et al. (2015); Amrhein et al. (2019) and Muff et al. (2022). Due to a relatively small dataset, all available data are presented whenever possible and narrative language of evidence is applied. Before the analysis, data were tested for normality and homogeneity of variances using Bartlett’s test. Since all data satisfied the assumptions for analysis of variance, a General Linear Model was employed to analyze the relationship between the factors (substrate type and year of cultivation as fixed factors) and the responses variables, and to show the presence of the possible interactions between the factors. Further, Fisher LSD *post-hoc* tests were applied. The analyses were conducted using MiniTab® Statistical Software program package (Release 17.2.1 Minitab Inc., State College, PA, USA).

## 3 Results

### 3.1 Physical and chemical characteristics of virgin materials

Physical and chemical properties of unused substrates are presented in **Tables 2**, **3**, respectively. Virgin defibrated wood fiber is characterized by more than two-fold lower dry bulk density than the traditionally used substrates (**Table 2**). Water on fresh and dry weight, water volume %, easy obtainable water and water buffering capacity are much lower in wood fiber compared to coir and peat (**Table 2**). On the other hand, wood fiber is characterized by the highest air volume % (83.5 at 10 cm and 90.7 at 100 cm, respectively) among the studied substrates (**Table 2**).

**Table 2:** Physical properties of virgin materials.

		C*	P	WF
Dry bulk density kg/m^3^		72.9	90.7	36.9
Water on fresh weight g H_2_O/100 g	10 cm	85.3	87.4	79.3
	50 cm	77.7	79.1	65.5
	100 cm	76.6	76.2	64.2
Water on dry weight g H_2_O/100 g	10 cm	582.2	692.8	383.8
	50 cm	349.4	379.4	189.6
	100 cm	327.3	319.5	179.7
Total pore volume ml/100 ml (humid 10 cm) TPV		95.4	95.0	97.6
Air volume % (ml air/100 ml fresh sub.)	10 cm	52.9	32.2	83.5
	50 cm	68.5	61.2	90.1
	100 cm	71.7	67.9	90.7
Water volume % (ml H_2_O/100 ml fresh sub.)	10 cm	42.5	62.8	14.1
	50 cm	26.7	33.9	7.2
	100 cm	23.7	27.4	6.9
Easy obtainable water		15.8	28.9	6.9
Water buffering capacity		3.0	6.5	0.4
Shrink %		12.6	12.1	<5.0
Moisture content % (g/100 g fresh weight)		73.1	66.2	46.7
Dry matter content % (g/100 g fresh weight)		26.9	33.8	53.3
Organic matter % (g/100 g dry weight)		95.8	64.8	99.7

*C, coir; P, peat; WF, wood fiber.

**Table 3:** Chemical properties of virgin materials.

		C*	P	WF
ORG. CARBON (OC)	%/DM	47.9	34.2	47.9
LIGNIN	%/OM	40.0	23.8	25.7
HEMICELLULOSE	%/OM	13.4	21.3	11.2
CELLULOSE	%/OM	38.3	40.0	52.6
Residual	%/OM	8.4	15.0	10.5
N	g/kg DM	4.43	7.55	0.64
C/N	-	108	45	749
P	g/kg DM	0.163	0.283	<0.05
K	g/kg DM	1.514	0.749	0.33
Mg	g/kg DM	0.621	0.996	<0.6
Ca	g/kg DM	5.67	15.67	<3.0
Na	g/kg DM	0.81	0.69	0.041
CEC	cmolc/kg DM	59.8	109.0	3.4
pH-H_2_O	-	6.49	6.59	4.41
EC	µS/cm	120	57	55
OUR (O_2_ Uptake)	mmol/kg OM/hr	1.5	1.5	2.6
CO_2_ Release	mol CO_2_/kg OM	0.32	0.30	2.19
N Immobilization	%	30.9	-4.4	22

*C, coir; P, peat; WF, wood fiber; NDF, Neutral detergent fibre; ADF, acid detergent fibre; CEC, cation exchange capacity; OUR, oxygen uptake rate.

Organic carbon content was identical in wood fiber and coir, and slightly lower in peat (**Table 3**). While hemicellulose levels are comparable in all three virgin materials (varying from 11.2% in wood fiber to 13.8% in peat), the cellulose fraction is much higher in wood fiber (52.4%) than in coir (36.7%) and peat (25.9%). Wood fiber seems to be the most inert material, with the lowest concentration of plant nutrients. Consequently, it has the lowest CEC and EC. This substrate had also the lowest pH (4.41 vs 6.49 and 6.59 for coir and peat, respectively). Typically for lignocellulosic materials, wood fiber is characterized by a high C/N ratio and a much higher CO_2_ release rate and oxygen uptake rate compared to peat and coir. Interestingly, the nitrogen immobilization essay revealed the highest values for coir (30.9%), followed by wood fiber (22%) and peat (-4,4%). Negative values observed in peat indicate nitrogen mineralization in this material (**Table 3**).

### 3.2 Physical and chemical characteristics of reused substrates

**Figure 1:**
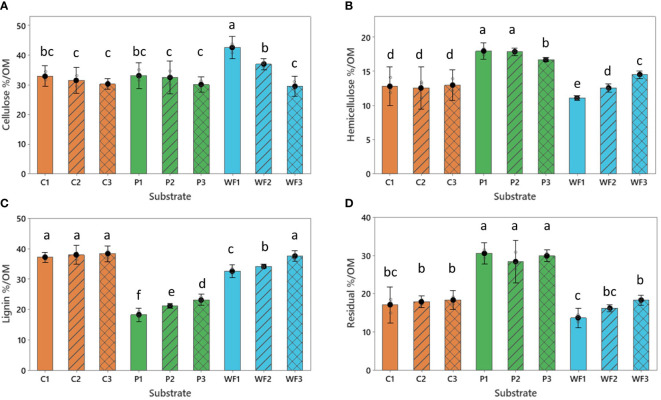
**(A)** Cellulose (%/OM), **(B)** hemicellulose (%/OM), **(C)** lignin (%/OM) and **(D)** residual fraction (%/OM) content in coir (C), peat (P) and wood fiber (WF) after one (1) two (2) and three (3) growing seasons. For all treatments n = 3, where n is one randomly selected tray representing each substrate type. Means that do not share a letter are significantly different based on the Fisher LSD Method (95% CI).

After the first year of production the highest cellulose percentage was detected in wood fiber, followed by coir and peat (**Figure 1A**). There was only weak evidence for decrease in cellulose content during consecutive cycles of coir and peat reuse. However, data revealed very strong evidence for decrease in cellulose percentage in wood fiber. After 3 growing cycles the cellulose content in this material was comparable to coir and peat. Overall, hemicellulose was highest in peat (**Figure 1B**). In both coir and peat, the percentage of hemicellulose was relatively stable across the age of substrates (**Supplementary Table 1**). The opposite situation was observed in wood fiber, where a rather low initial hemicellulose rate increased throughout all years (**Figure 1B**). For the lignin percentage in the substrate, the lowest levels were observed in peat, while the highest were in coir and wood fiber. In general, during the reuse cycles, the total percentage of lignin increased in peat and wood fiber and remained stable in coir (**Figure 1C**). Residual organic matter percentage was the highest in peat. Both coir and peat revealed relatively stable levels of residual organic matter, while a significant rise was noted in wood fiber (**Figure 1D**).

**Figure 2:**
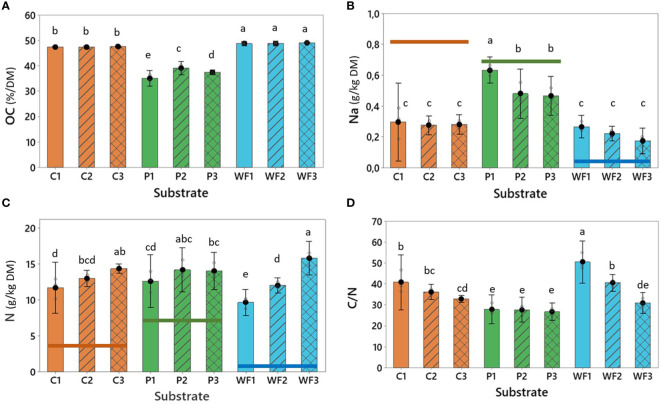
**(A)** Organic carbon (OC, %), **(B)** Total sodium content (Na, g/kg DM), **(C)** Total nitrogen content (N, g/kg DM) and **(D)** C/N ratio in coir (C), peat (P) and wood fiber (WF) after one (1), two (2) and three (3) growing seasons. Horizontal lines represent nutrient levels observed in unused materials. For all treatments n = 3, where n is one randomly selected tray representing each substrate type. Means that do not share a letter are significantly different based on the Fisher LSD Method (95% CI).

The lowest percentage of organic carbon was observed in peat and was comparable to unused virgin material and after three years of production. However, a slight increase was observed after the second and third year of production (**Table 3**, **Figure 2A**). On the other hand, both unused and reused wood fiber and coir had stable percentages of organic carbon (**Table 2**, **Figure 2A**). Relatively high content of sodium (Na), which is a potentially harmful element, was observed especially in virgin coir and peat (**Table 3**). However, decrease of Na in those substrates was noted after the reuse (**Figure 2B**). Accumulation of N was observed in all substrates when compared to N contents in virgin materials (**Table 3**). Across the three analyzed growth cycles, the most rapid N accumulation was noted for wood fiber, resulting in concentrations comparable to or higher than those observed in peat and coir (**Figure 2C**). Consequently, the C/N ratio of wood fiber, which has the highest values across the substrates after one year of production, after two and three years of use drops to the levels observed in substrates traditionally utilized in SCS strawberry cultivation (**Figure 2D**).

**Figure 3:**
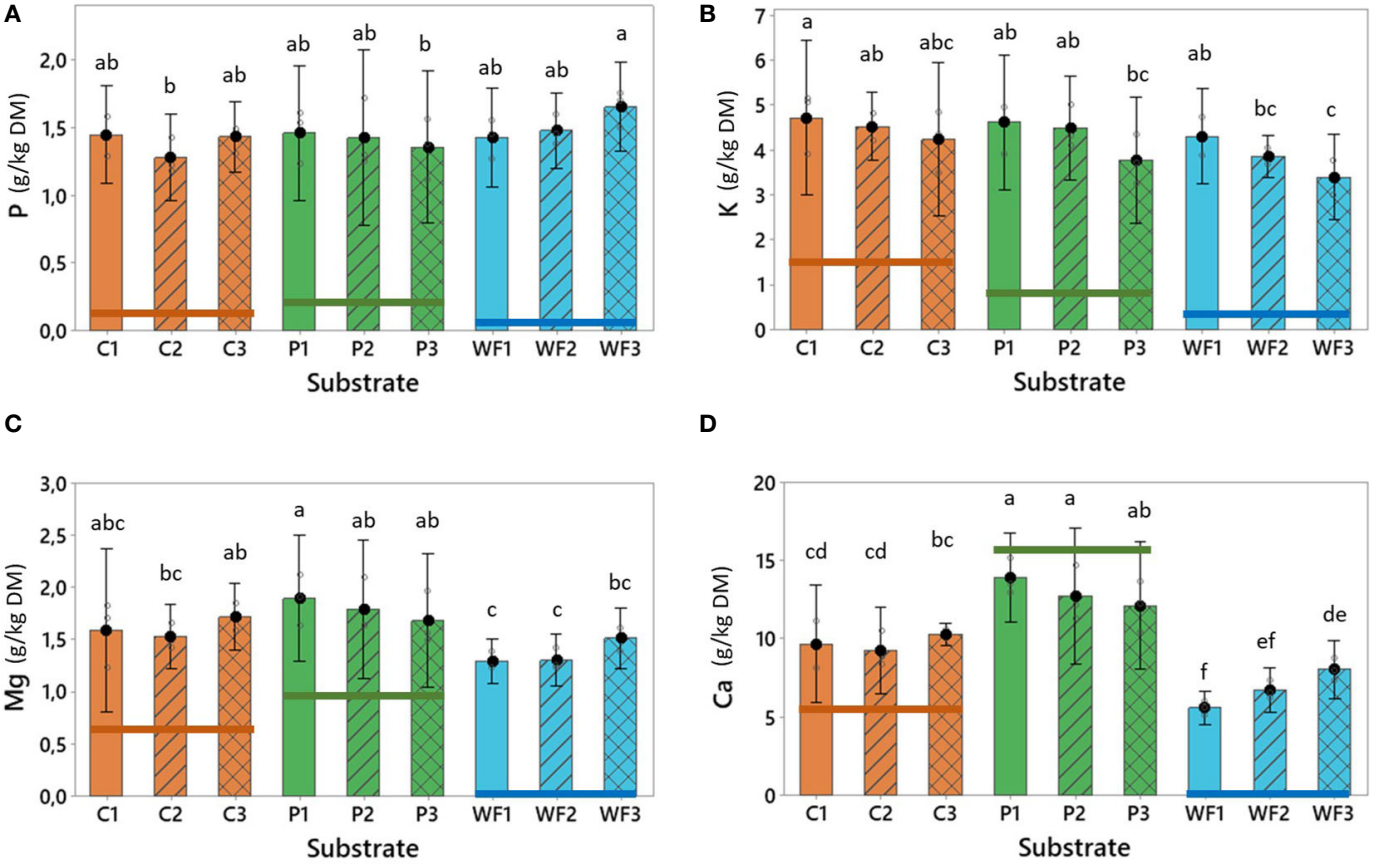
**(A)** Phosphorus (g/kg DM), **(B)** Potassium (g/kg DM), **(C)** Magnesium (g/kg DM) and **(D)** Calcium (g/kg DM) content in coir (C), peat (P) and wood fiber (WF) after one (1), two (2) and three (3) growing seasons. Horizontal lines represent nutrient levels observed in unused materials. For all treatments n = 3, where n is one randomly selected tray representing each substrate type. Means that do not share a letter are significantly different based on the Fisher LSD Method (95% CI).

Mineral composition of spent growing media is highlighting the trend in nutrient accumulation during the repetitive production years in various substrates (**Figure 3**). While unused substrates are highly different in mineral composition (**Table 2**), such contrasts decreased after the first production cycle (**Figure 3**). Compared to the virgin materials, the spent growing media were clearly higher in N, P, K, and Mg while Ca contents were slightly lower in the spent peat. P content was similar and stable in all spent materials and was not affected by age (**Figure 3A**, **Supplementary Table 1**). On the other hand, K accumulated to comparable levels in all materials after the first year of production and then decreased when the substrates were further reused. The highest decrease in K content was observed for wood fiber (**Figure 3B**). The greatest levels of Mg were detected in peat, while lower in coir and wood fiber (**Figure 3C**). Peat had the highest ability to accumulate excess Ca, and the levels were relatively stable across production years (**Figure 3D**). On the contrary, wood fiber was characterized by the lowest ability to accumulate Ca, however, during the consecutive production cycles the increase in Ca contents was significant and finally similar to values observed in coir (**Figure 3D**).

Approximation of the amount of macronutrients incorporated into a production system through the reuse of spent growing media show that the supply is affected by both substrate type and number of reuse cycles (**Table 4**). Under the applied fertigation strategy, coir, peat, and wood fiber after the first production cycle provide to the second production cycle ca. 90, 117 and 38 kg of N/ha, respectively, and those values increase by ca. 10kg after each of reuse (**Table 4**). The amount of P, K and Mg accumulated in the spent substrate per hectare of production is ca. 7-10 times lower than N, being the highest in peat and the lowest in wood fiber. While the content of P and Mg was relatively stable in each substrate across the reuse cycles, K revealed slight depletion (**Table 4**, **Supplementary Table 2**).

**Table 4:** Approximation of the amount of macronutrients provided with spent growing media (in kg/ha of table-top strawberry production).

	N (kg/ha)	P (kg/ha)	K (kg/ha)	Mg (kg/ha)
C1*	93 c	11 a	37 abc	13 c
C2	103 bc	10 ab	36 abc	12 c
C3	114 abc	11 a	34 c	14 bc
P1	117 ab	14 a	43 a	17 a
P2	130 a	13 a	41 ab	17 a
P3	130 a	13 a	35 bc	16 ab
WF1	38 e	6 c	17 d	5 d
WF2	48 de	6 c	15 d	5 d
WF3	63 d	7 bc	13 d	6 d

*C, coir; P, peat; WF, wood fiber; 1, 2, 3 – years of reuse.

Means that do not share a letter are significantly different based on the Fisher LSD Method (95% CI).

### 3.3 Plant performance, berry yield and quality

**Figure 4:**
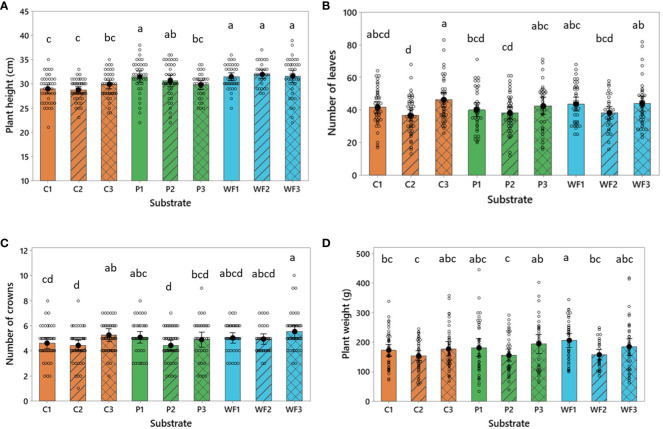
**(A)** Plant height (cm), **(B)** number of leaves **(C)** crowns and **(D)** fresh weight (g) of aboveground plant biomass (g) in strawberries grown in coir (C), peat (P) and wood fiber (WF) after one (1), two (2) and three (3) growing seasons. For all treatments n = 48. Each dot represents one plant. Means that do not share a letter are significantly different based on the Fisher LSD Method (95% CI).

All plants were visually assessed, and no visible symptoms of any stressful conditions were recorded. However, variability in the plant material was very high (each dot in **Figure 4** represents parameters of an individual plant). In general, the highest strawberry plants were recorded in wood fiber independently of the age of substrates. Comparable performance was observed in plants grown in new peat; however, reused peat tended to produce shorter plants. On the other hand, the lowest strawberry plants were observed in coir, **Figure 4A**). Number of leaves, crowns and plant weight are plant architectural parameters which are usually correlated with plant height, and a similar trend was observed here (**Figures 4B-D**). Number of leaves and crowns are comparable across the treatments; nonetheless, there is a visible tendency that strawberry plants were more vegetative when grown in substrates reused three times (**Figure 4B**). Plant biomass production varied greatly but was generally maintained, even though it was slightly reduced in the substrates used for two years (C2, P2, WF2; **Figure 4D**, **Supplementary Table 3**).

**Figure 5:**
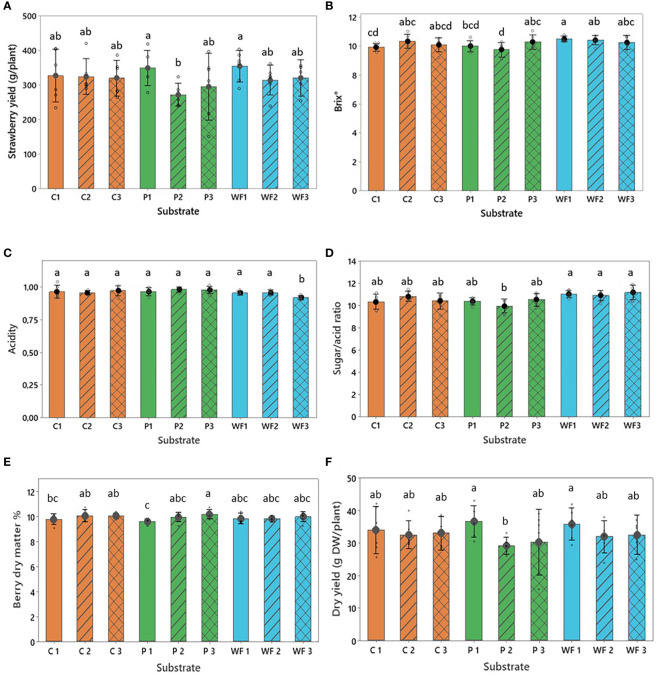
**(A)** Total yield of marketable berries (>28mm), **(B)** soluble solids (°Brix), **(C)** acidity (% of citric acid), **(D)** ratio between sugars and acids, **(E)** berry dry matter (%) and **(F)** dry yield (g DW/plant) grown in coir (C), peat (P) and wood fiber (WF) in one (1), two (2) and three (3) growing seasons. For all treatments n = 6, where n is the average of eight plants from the same repetition. Means that do not share a letter are significantly different based on the Fisher LSD Method (95% CI).

In general, strawberry yield was satisfactory and comparable across all investigated substrates (**Figure 5A**). A slightly higher yield was observed in plants from peat and wood fiber in the first year of production compared to coir. Yield from plants in coir, however, remained relatively stable across reused substrates while for plants in peat and wood fiber weak evidence for reduction of their yielding potential in the second (p<0.008 for peat) and third year of production in the recycled substrates is present. However, the reduction was not large, and the plants performed similarly to those grown in coir with no statistical differences observed (**Figure 5A**). Dry matter of the berries was also relatively stable across the substrates (**Figure 5E**), increasing only slightly in berries grown in peat used for the third time (**Figure 5E**). Berry dry yield, obtained by the recalculation based on dry matter content (**Figure 5E**) revealed a pattern parallel to the fresh weight yield (**Figures 5A, F**).

Comparison of the means suggests a tendency for higher sugar accumulation in strawberries grown in wood fiber (**Figure 5B**, **Supplementary Table 3**) and reduced berry acidity in this substrate (especially in the variant reused twice, **Figure 5C**) consequently resulted in strawberries with a slightly better taste (sugar/acid ratio ca. 0.5 to 1 point higher in WF than in C and P, **Figure 5D**). However, a high variability in the sugar/acid ratio (strawberry taste) in a given plant didn’t allow to detect very strong statistical evidence for differences between the growing media age (**Figure 5D**, **Supplementary Table 3**).

## 4 Discussion

### 4.1 Sustainability

Worldwide, large quantities of spent, nutrient-rich growing media residues are disposed, not being recycled. It is suggested that circular economy (CE) strategies for materials used in agricultural production include reuse within the same or between different value chains. This would minimize waste generation and decrease the need to incorporate new materials (Sayadi-Gmada et al., 2019; Circle Economy, 2021). Despite of the observed changes in chemical properties of the substrate after a production cycle, the results here show that all the tested growing media (both traditional, like peat and coir, and a novel substrate candidate, stand-alone wood fiber) can be successfully reused in SCS strawberry production. A similar observation was made by Baevre and Guttormsen (1984) when tomato and cucumber produced in reused peat bags was analyzed. Shuttleworth et al. (2021) proved the potential of SCS strawberry production in reused coir. Beyond that, the present study indicates that the stand-alone wood fiber, previously investigated as a potential raw material which can replace peat and coir (Woznicki et al., 2023) can also be utilized in consecutive growing cycles without significant deterioration or adverse effect on yield. This provides possibilities to fulfill the potential of this material for economic and environmental benefits in a CE approach as well as being an important step toward broader implementation of “lean production” paradigm (Gruda, 2019; Dahmani et al., 2021; Velasco-Muñoz et al., 2022).

### 4.2 Nutrient dynamics

During the reuse of substrates, some nutrients can be accumulated (sometimes reaching the toxicity levels) in the material, and further transferred into newly established plants and utilized as a start fertilizer (Vandecasteele et al., 2018b; Aurdal et al., 2023). Also, in the present study, this phenomenon was observed, however, any excess accumulation of salts, including sodium, was not noted (**Figure 3**), indicating that the utilized fertigation strategy was adequate and did not cause any serious salt stress or excessive nutrient retention in any of the substrates. This was reflected in the biomass production and overall, well-being of the plants (**Figure 4**). The amount (kg/ha) of nutrients transferred into the next production cycle by the spent growing media (**Table 4**) was generally parallel to the range observed by Vandecasteele et al. (2023) in greenhouse strawberry production. However, in the present study a higher accumulation of K in comparison to Mg was observed, while Vandecasteele et al. (2023) noted the opposite trend. This might be due to fertigation strategy implemented by Vandecasteele et al., 2023, which had almost identical ratios between N, P and K as those implemented here, but provided approximately two times higher levels of Mg (See: Materials and Methods section).

Interestingly, wood fiber showed the highest and most rapid nitrogen accumulation, and therefore, might be the reason for the most vigorous growth (plants were the highest among all tested substrates, **Figure 4**). Consequently, the observed C/N ratio also dropped rapidly, and after three years of production, the used wood fiber was characterized by a C/N ratio comparable to peat. This also indicates that microbial nitrogen immobilization, which can be a serious problem for plants grown in wood fiber-based substrates (Harris et al., 2020) was avoided by a precise adaptation of the fertigation strategy with slightly higher EC and more dense distribution of drips in comparison to common practice (Aurdal et al., 2023; Woznicki et al., 2023). A trend with decreasing potassium (K) accumulation during reuse of substrates (the highest in wood fiber) agrees with Vandecasteele et al. (2018b); Vandecasteele et al., 2023), and might be explained by the fact that strawberry has a relatively high demand for K. It is known that during fruit ripening, fruits represent the largest sink for K and N (Tagliavini et al., 2005). The stand-alone wood fiber substrate is characterized by highest porosity and lowest CEC of the tested substrates (**Tables 2**, **3**) and therefore substantial leakage of nutrients should be compensated. Thus, it can be hypothesized that an updated fertigation strategy for wood fiber should include additional source of K to satisfy the needs of high yielding cultivars. It is worth mentioning that the applied fertigation strategy was also suitable for the plants grown in peat and coir, indicating plasticity and adaptation ability of strawberry plants when grown in an organic substrate.

### 4.3 Quality attributes

The sugar – acid ratio is a broadly used parameter for indication of strawberry taste and consumer acceptance (Li et al., 2022) and may be a proxy for a sensorics panel evaluating strawberry attractiveness. This parameter showed constantly the highest values in strawberries grown in wood fiber (**Figure 5**). It was the consequence of a relatively high sugar accumulation and simultaneously, stable acidity of the studied berries, with exception of berries from plants grown in the oldest wood fiber substrate, which reduced acidity. This observation agrees with the previous study, where strawberry plants grown in wood fiber-based substrate revealed a slightly higher sugar accumulation (Woznicki et al., 2021). For optimal aerobic respiration of the roots, sufficient oxygen level in the root zone is crucial. Anaerobic conditions in the root zone can inhibit uptake of nutrients as well as cell division rate (Soffer et al., 1991; Rocksch et al., 2008). Due to higher porosity (Woznicki et al., 2023) and much higher air volume % (**Table 3**) of wood fiber, strawberry plants are less likely to experience stressful anaerobic conditions. This fact might be partially responsible for the increase in sugar accumulation in berries as observed in the present study.

### 4.4 Substrate stability

Microbial degradation is a common process that affects all organic-based growing media. When combined with shrinkage and swelling, these factors can lead to negative consequences for the structural stability of the media (Jackson et al., 2009; Carlile et al., 2015). In this study, the degradation process was most visible in stand-alone wood fiber (**Figure 1**) where the percentage of cellulose dropped to the levels observed in coir and peat. In contrast, coir and peat revealed relative stability across production cycles (**Figure 1**). The degree of stability loss is mainly affected by the chemical composition. Peat exhibits high resilience against decomposition, due to the fact that *Sphagnum* mosses incorporate lignin-like polymers into the cell walls, which plays a pivotal role in the resistance to degradation (Prasad and Maher, 2004). Moreover, the unique pectin-like substances, such as sphagnan, found in the cell walls of *Sphagnum* mosses (Stalheim et al., 2009), contribute to their durability, as suggested by Hájek et al. (2011). The resistance to degradation seen in coir can be attributed to the intricate composition of lignocellulose complexes, comprising cellulose, hemicellulose, and lignin, which are interlinked through both covalent and non-covalent bonds. These bonds confer significant resistance against degradation (Carlile et al., 2015). On the other hand, the biological degradation susceptibility of conifer wood fiber can be especially attributed to the high decomposing vulnerability of cellulose and hemicellulose (Fengel and Wegener, 1989). In the present study, the observed change in hemicellulose (and to lesser extent lignin) percentage in wood fiber (**Figure 1**) is likely related to the proportional decrease in percentage of cellulose which led to an overall increase of percentage share of other constituents.

Despite high variability in plant architecture within each treatment, there is an apparent trend that plants grown in substrates used for a second production cycle had slightly reduced number of leaves, crowns, and overall biomass production (**Figure 4**). This phenomenon might be explained by the influence of the planting strategy. Plugs planted in substrates used for two years were planted next to the old plugs’ residuals, while in the substrates used for the third time, half of the old plugs were removed, and new ones were planted instead. The differences in local nutrient distribution and space for root development might be responsible for this unexpected observation. However, it can be concluded that the placing of new plants in the old substrate had only marginal effect on strawberry yield.

As shown in the present study, it is possible to grow strawberries in reused peat, coir, and wood fiber without any pretreatment. However, during the scenario where rootzone diseases are present, the reuse of substrate can become a risky strategy which can lead to great economic losses. Therefore, it can be suggested that steam sanitation, which eliminates pathogens and weeds, should be applied whenever the risk of pathogen or weed contamination is serious (Vandecasteele et al., 2020). Recently, a successful eradication of powdery mildew from strawberry transplants was obtained using aerated steam (Stensvand et al., 2023). It can be hypothesized that the same equipment utilizing elevated temperature of treatment can be a promising tool for effective substrate sanitization.

The reuse of growing media reduces the costs of purchase of new blends and the gate fee for waste collection for the grower. The net return of reusing growing media is strongly dependent on the price of virgin growing media. Spent growing media can be directly reused for another strawberry cultivation, without negative impact on yield or fruit quality. When destined for direct reuse by the grower, the need for sanitation needs to be assessed by the grower based on experiences and observations during the previous growing cycle. Sanitation is necessary whenever spent growing media are transferred from one grower to another, to avoid any risks related to weeds or pests/diseases.

## 5 Conclusion

This work evaluated the feasibility of substrate reuse for SCS strawberry production. Two commonly used substrates, peat and coir, as well as a novel alternative, wood fiber from Norway spruce, revealed potential for successful implementation even after two cycles of production. Wood fiber revealed an ability for nutrient accumulation. Its chemical composition changed, making it more similar to the commonly used growing media utilized for SCS production. Therefore, it can be concluded that not only peat and coir, both commercial standards with sustainability issues, but also wood fiber, a more sustainable alternative, can be successfully reused, ensuring satisfactory yields of high-quality strawberries.

## Data availability statement

The original contributions presented in the study are included in the article/**Supplementary Material**. Further inquiries can be directed to the corresponding author.

## Author contributions

TW: Conceptualization, Data curation, Formal analysis, Investigation, Methodology, Validation, Visualization, Writing – original draft, Writing – review & editing, Funding acquisition, Resources. KK: Conceptualization, Data curation, Formal analysis, Funding acquisition, Investigation, Methodology, Resources, Validation, Writing – original draft, Writing – review & editing. BV: Conceptualization, Data curation, Formal analysis, Investigation, Methodology, Resources, Validation, Writing – review & editing. AS: Conceptualization, Funding acquisition, Investigation, Methodology, Project administration, Resources, Supervision, Writing – review & editing.
