# Supplementary material for: Reuse of coir, peat, and wood fiber in strawberry production
Source: Front Plant Sci. 2024 Jan 12;14:1307240. doi: 10.3389/fpls.2023.1307240 (PMC10811249; doi:10.3389/fpls.2023.1307240)
Supplement: Supplementary file 1 [file DataSheet_1.pdf]

Supplementary materials for article titled: **Reuse of coir, peat, and wood fiber in strawberry production.**

Supplementary Table S1. P-values obtained by Analysis of Variance (ANOVA) for chemical parameters of spent growing media across analyzed substrate types, year of cultivation and their interactions.

Substrate parameters	Cellulose %/OM	Hemicellulose %/OM	Lignin %/OM	Residual %/OM	OC (%)	Na (g/kg DM)	N (g/kg DM)	C/N	P (g/kg DM)	K (g/kg DM)	Mg (g/kg DM)	Ca (g/kg DM)
Substrate type	0.000	0.000	0.000	0.000	0.000	0.000	0.083	0.000	0.233	0.041	0.001	0.000
Year of cultivation	0.000	0.081	0.000	0.015	0.000	0.003	0.000	0.000	0.571	0.019	0.604	0.537
Substrate*Year	0.000	0.000	0.003	0.010	0.000	0.098	0.005	0.000	0.419	0.912	0.386	0.045

Supplementary Table S2. P-values obtained by Analysis of Variance (ANOVA) for nutrients accumulated in substrate per hectare of strawberry production across analyzed substrate types, year of cultivation and their interactions.

Nutrient accumulation in substrate per ha	N (kg/ha)	P (kg/ha)	K (kg/ha)	Mg (kg/ha)
Substrate type	0.000	0.000	0.000	0.000
Year of cultivation	0.000	0.736	0.045	0.813
Substrate*Year	0.505	0.661	0.820	0.517

Supplementary Table S3. P-values obtained by Analysis of Variance (ANOVA) for plant architecture and berry quality parameters across analyzed substrate types, year of cultivation and their interactions.

Plant architecture and berry quality	Plant height	No. of leaves	No. of crowns	Plant weight	Berry yield	DM %	Dry yield (g/DM)	Brix°	Acidity	Sugar/Acid
Substrate type	0.000	0.605	0.061	0.327	0.406	0.782	0.713	0.021	0.004	0.000
Year of cultivation	0.821	0.001	0.004	0.003	0.086	0.025	0.060	0.886	0.650	0.675
Substrate*Year	0.054	0.640	0.388	0.572	0.589	0.480	0.710	0.063	0.074	0.170
